# Senile Hyperthrophy of the Prostate, with Cases

**Published:** 1874-04

**Authors:** James S. Bailey

**Affiliations:** Albany, N. Y., Formerly of Alabama but more recently from Hempstead, Texas


### SENILE HYPERTROPHY OF THE PROSTATE, WITH CASES.

BY JAMES S. BAILEY, M.D., OF ALBANY, N. Y.,
Formerly of Alabama but more recently from Hempstead, Texas.

In entering upon the consideration of the Hypertrophied state of the prostate gland in those advanced in age, it is highly important to know, by actual observation, the appearance of the organ in its normal condition.

Writers differ materially in regard to this, and it is evident that the results which have been obtained, have not been based upon observations sufficiently extended and numerous to settle the question of measurement and weight of different organs.

When the size deviates inconsiderably, it is apparent that the anatomical relations must of necessity be deranged. The exact age, laws and circumstances, which subject the prostate to this morbid change seem to be but little understood.

The adult prostate is commonly described as resembling a full-grown chestnut in form and size, and as ranging in weight from 4 to 6 drachms. According to a tabulated statement given by Sir Henry Thompson, in 33 normal prostates, 5 drachms and 33 grains is given as the heaviest, and 3 drachms and 34 grains as the lightest weight. The average weight is estimated at 4 drachms and 38 grains, so the usual weight may be estimated safely at from $4\frac{1}{2}$ to $4\frac{3}{4}$ drachms.

Dr. Messer, of the Royal Naval Hospital, Greenwich, in one hundred dissections of prostates, taken from subjects at or over sixty years of age, arrives at nearly the same result, and gives 4 drachms and 57 grains as an average weight from forty-five preparations taken from subjects from sixty to ninety-four years old.

The measurement of the prostate also differs much through the transverse diameter; it exceeds in the majority of cases the antero-posterior diameter by a fifth or a sixth.

Sir Henry Thompson presented the result of his investigation in the dissection of fifty adult cases to the Medico-Chirugical Society, thirty-three were healthy and were as follows:

The measurement from the base to the apex ranged between 1.3 and 1.8 inches. The most common measurement was 1.4 inches. The most common transverse measurement 1.75 inches. The most common measurement of thickness was 0.7 inches. These nearly correspond with the researches of Gross, Hodgson, Senn and Deschamps.

Hypertrophy of the prostate is quite common in men advanced to the age of fifty or sixty years, though it does not necessarily follow that in every instance it should be found. The enlargement gradually takes place and obstructs eventually the free passage of urine from the bladder.

This enlargement may not be due to inflammatory action of the gland itself or from fibrinous deposits, but to tissue increasing in its formation, which finally constitutes hypertrophy.

The exact increase of weight necessary to constitute hypertrophy is difficult to define, but a prostate weighing seven drachms is usually considered an abnormal condition—a hypertrophy.

The first indication of hypertrophy is a sense of fullness in the parts, and if a section could now be made through the anterior commissure so as to lay open the urethral passage longitudinally, the lateral lobes would appear fuller than natural so that they would press upon each other.

The enlargement of either lateral lobe may be uniform, either lobe may predominate, or the median portion may be largely developed. When this is the case with either lateral lobe, the urethra is by pressure diverted from its natural course.

The most usual result of enlargement is to obstruct the flow of urine, although cases occur in which the bladder is unable to retain the urine and it is voided involuntarily.

If obstruction is the most common cause of enlargement, it is highly important to know the changes produced in the size and course of the urethra, in order to know how to be able to effect relief by mechanical means, by the passage of a catheter.

The first effect is to increase the antero-posterior diameter of the prostatic urethra, and also, diminution of the lateral and transverse diameter, so that the canal becomes narrowed and perhaps tortuous. The length of the prostatic urethra is also increased by this form of enlargement. It is sometimes elongated from its normal length 1¼ inch to perhaps 3 inches. Each form of enlargement causes a change in the natural direction of the urethra. The enlargement of the median portion shortens the curve; the same is found to be the case when one lateral lobe is unduly enlarged, which gives a reflection or lateral curve to the course of the urethra, and vice versa.

The symptoms in the earliest stages of hypertrophy of the prostate are not sufficiently marked to attract attention of the patient. In fact, in this intermediate stage, a period of several months to that of several years may intervene before anything unusual may be observed in the act of micturition, caused by the organic change of this gland.

The constitution of the patient and the character of the enlargement may influence much the manifest symptoms, for in some instances we find inconsiderable enlargement producing grave symptoms and in others very considerable enlargement may take place with little inconvenience.

The following symptoms are usually observed as making their appearance: If complete retention takes place from exposure from cold, one of the earliest symptoms is a diminution in the force of the ejected stream of urine, and when the effort is made to expel it an uncertainty and hesitation to flow is experienced. The size of the stream may not be diminished, but its force cannot be much augmented by an additional effort. In some cases of enlargement any additional straining to void urine only increases the difficulty. The desire to micturate is often increased, and the relief afforded by the act is less complete and the ordinary satisfaction is not experienced after emptying the bladder; the same desire after a few minutes is again manifest, which is often repeated without relief.

The odor of the urine is often disagreeable to the patient, and there is a sense of fullness and uneasiness experienced about the rectum and perineum. As the necessity to expell, the water becomes more frequent, the vigorous effort causes more or less irritation about the prostate, and it is not uncommon for defæcation to accompany that of micturition, and for hemorrhoidal protrusions and swellings to result as a consequence in an effort to obtain relief.

As the disease advances, pain is developed in the groins and thighs and sometimes is transferred to the testicles. The pain is also directly associated with the condition of the bladder, which is attested by the constant aching and gnawing sensation about the pubes, consequent upon a distended bladder associated with chronic inflammation of that organ. The urethra also sympathizes and is manifested by a smarting soreness, which frequently extends to the glans penis with much acuteness, and it is not unfrequent for the testicle to become swollen and tender, and from the vascular excitement of the parts frequent erections of the penis will take place. From the gradual wear on the constitution in the effort to relieve the bladder by micturition, it is evident that the disease is steadily increasing as the signs of progress are unmistakable. In rare cases the growing prostate allows the urine to escape involuntarily, and the viscus is unable to contain but a small quantity of urine. In the large majority of cases the effect is far different, and is to close the urethro-vesical outlet, which requires a preternatural effort to expel the fluid without the aid of the catheter; the result being the act never takes place except when the distention adds weight sufficient to assist in expelling the urine, while a certain quantity remains, retaining all below a certain level; and if unrelieved by art the evil must eventually tell upon the constitution.

When an overflow takes place and the urine dribbles away drop by drop, wetting the patients clothing and exhaling a moist offensive odor, it is exceedingly annoying to the patient and adds to his personal sufferings, for in addition to his own conciousness of his trouble he is aware that every one around him must also participate in the offensiveness of the foul smell.

When the bladder is distended there is always dullness upon percussion above the pubes and a tumor may be plainly felt. When the act of micturition becomes so difficult, the patient is often compelled to assume the most favorable position for this purpose, which is to lean forward and support his head against some object, in order to make the urethro-vesical orifice the most depending portion of the bladder; his legs are also extended widely to support him in this powerful, though perhaps fruitless effort to void urine. An idea of the physical force requisite may be conveyed from the fact that hernial protrusions are sometimes caused by the effort.

The functions of the kidneys also become embarrassed from the over distention of the bladder, and the urine is backed up through the uretus and uremic, poisoning is thereby induced which is followed by fatal consequences.

In noting the characteristic appearance of the urine important changes can be discovered; it ceases to be transparent and is cloudy and of a greenish tinge, or pale with shreds of suspended floculi. The mucous deposits float upon standing, with an opaque or iridescent deposit upon the surface. In the latter and advanced stages the mucous increases in quantity and appears tenaceous and stringy and adheres to the sides of the vessel, and when poured out flows in a mass; in addition to this, sometimes an opaque and creamy deposit which is pus mixed with tripple phosphates is also found.

The odor is pungent and ammonical and sometimes extremely fœtid. The chemical reaction is at first neutral, afterwards alkaline varying intensely. The specific gravity is often very high.

Microscopically may be observed blood and pus, corpuscules and epithelium. Among the crystals may be found the triple phosphates of magnesia or ammonia in a variety of sizes and form, besides granular matter; crystals of uric acid and oxalates may also be found. Albumen is usually absent unless introduced by blood or pus, unless the kidneys are invaded by disease, by inflammation or by regurgitation of the urine from the bladder, or from pressure interfering with the secretions then uriniferous tubes and albumen will be deposited in the urine.

The cause of hypertrophy must necessarily be obscure, though some are more plainly defined than others. The following may be noted as among the most prominent: Habitual congestion upon account of obstruction of vessels contributing to the circulation of the prostate. Horseback exercise produces congestion from bruising upon the saddle. Instances are common when gentlemen exercise much in this way.

Sir Astley Cooper regards the enlargement as a consequence of age and not of disease. Sir Benj. Brodie regards it a phenomena which marks the decline of life, for says he, when the hair turns gray and earthy matter begins to be deposited in the coats of the arteries the prostate usually begins to enlarge.

Dr. Gross attributes its induction to repeated sextual intercourse and from irritation from vesical calculus, to frequent use of stimulating diuretics, to alcoholic drinks, exposure to cold, gout and rheumatism, external violence from introduction of the catheter, straining at stool, from chronic diarrhœa, etc.

Desault thinks that repeated attacks of gonorrhœa may frequently cause it.

In youth the prostate becomes enlarged by interstitial plastic effusion the result of inflammatory action. In age there is an unnatural developement of the gland itself.

In the treatment of retention of urine from enlarged prostate the first indication is to overcome the internal congestion, and to allay pain and quiet the involuntary disposition to pass water; second, to relieve the over distention of the bladder by giving free exit to the urine if possible through the urethra by means of a catheter.

Before catheritization is resorted to, the employment of a hot bath to the hips with a temperature of 100°, at least, in many cases, is very efficient and is beneficial in almost every stage, and is not accompanied with any risk of injury to the parts, but prepares the parts for the use of the catheter. The external warmth directs the blood to the surface filling the external vessels and diverting the blood from the deeper seated vessels.

Opium is next indicated and if the bath does not relieve it may be given in full doses. If these means are not effective it is best to resort without delay to the use of the catheter. This procedure requires very great caution and cure in order to keep the instrument in the urethral canal.

Sir Henry Thomsan remarks that in the early part of his practice he preferred the silver catheter, but now with more experience he prefers the gum-elastic catheter.

The silver catheter has been found to be the most serviceable in my hands, although it would be well for the surgeon to be provided with both. The larger sizes will usually be the best, certainly as large as No. 10, and in length it should be 14 inches, its curve should comprise one-fourth of a cirle measuring from 4 to 5½ inches in diameter.

In manipulating with the catheter, by way of introduction, the instrument should be held with extreme lightness and delicacy, or the operator may fail to appreciate any resistance if encountered at the point. The gum-elastic catheter sometimes, by the application of force, becomes bent and doubled within the urethra and disappears under pressure without accomplishing the desired end which the practiced hand readily detects.

When the urethra is irritable and liable to spasm, by holding the instrument gently against the strictured part, it soon ceases and the instrument then glides easily through.

In order to avoid making a false passage, as large an instrument as the urethra will admit should be employed, it should have a blunt point, and by gentle pressure it should be kept close to the upper surface of the urethra. The obstruction is usually encountered upon the lower part of the canal. Upon arriving at the pubic curve the handle should be depressed which elevates the point, and should it not enter readily the index finger should be oiled and introduced per anum, and by gentle pressure upward the catheter is elevated and pressed into the bladder.

Another catheter of recent invention deserves mention in relieving distention from hypertrophied prostate. I refer to the vertebrated prostatic catheter devised by Dr. Squires, of Elmira, N. Y.

It having been examined by most of you, I need only say that the curve is composed of sections of a quarter of an inch each, and dovetailed as it were into each other like the vertebra of fish, and through the center of the instrument are links of wire soldered together and connected to a wire traversing the catheter with a thumb-screw attached to the free end, by which the instrument may be made to assume and retain any desired curve.

It is claimed by surgeons that in tortuous passages created from unequal enlargements of the lobes of the prostate, that the joints of this instrument will traverse with greater facility than either the silver or gum-elastic catheter. Not having had personal nor practical experience myself with it, I shall leave it to surgeons whom I see present to praise its merits.

After having with considerable difficulty succeeded in introducing the catheter, the question arises whether it should be allowed to remain in position?

There is also a diversity of opinion in this respect, some deny the correctness of this view, and others insist that it should be left in position. It is considered by some to add to the irritability of the urethra already existing by its presence.

If it is decided that it should be left in position, which is best under some circumstances, it should not project but a short distance through the urethra, barely sufficient to allow the urine to escape lest it might cause irritation in the bladder. The gum-elastic catheter is usually preferred for this purpose. A plug should be inserted or some tubing attached to conduct the urine to an adjacent vessel.

Case I. Wm. Smith, a strong and powerfully built man, aged 72, machinist, took cold and found difficulty in urinating. July 7th, he was entirely unable to do so. Dr. Wm. H. Bailey and myself were called, and with difficulty succeeded in introducing a catheter. About 3 pints of dark, bluish, bloody urine escaped, which only partially relieved the pain, which he described as being across the loins and shooting down his thighs. It was with difficulty that he could get in and out of bed or change his position while in the recumbent posture.

His urine remained tinged with dark blood until the 22d of July, when we failed to empty his bladder, owing to the eyes of the catheter (which were small) filling with dark decomposed fœted blood.

Dr. Swinburne was then consulted, and by the use of a No. 11 silver catheter, with large eyes, the thick bloody urine was evacuated, and then by means of a fountain syringe attached to the catheter the bladder was washed out alternately with warm and cold water. Upon the next catherization the urethra seemed shortened more than an inch, and the urine escaped clear with occasionally a coagula of blood. About this time he manifested symptoms of uræmic poisoning; was delirious and spasmed; these symptoms gradually improved for one week after washing out the bladder twice each day. At the expiration of this time his appetite began to fail, and he finally succumbed to the disease, August 3d. Necropsy August 5th, 1872. Sectio cadaveris 24 hours after death. Rigor mortis marked. Abdomen—liver somewhat enlarged, edges rounded, ductus communis choledicus occluded; gall bladder small, containing a few calculi and a drachm of mucous; pancreas thickened and quite hard on section; normal in appearance; spleen healthy. Kidneys—right kidney healthy; left kidney the supra renal capsule enlarged to the size of the kidney itself, presenting on section a granular look and of a pale color; had the appearance of a morbid growth, kidney itself disorganized with only two or three pyramids remaining; ureters not affected. Bladder—walls considerably thickened; half an inch from the mouth of the urethra a longitudinal elevation an inch or more high, composed of the hypertrophied third lobe of the prostate gland, so situated as to fall as a valve across the mouth of the urethra. It was congested, was soft and rather friable and contained two or three perforations. Prostate enlarged to two inches, anterio-posterior diameter, and a little more than two inches in the transverse diameter. Urethra normal throughout, except at the external orifice where there was stricture and dense tissue; alimentary canal normal; thorax not examined.

July 11th—Urine examined and found alkaline, of low spec. gr., containing an abundance of albumen and blood globules on microscopical examination.

July 25th—It was again examined and found strongly alkaline and of ammoniacal odor, with a slight amount of albumen and depositing triple phosphates and phosphate of lime.

Through the kindness of Dr. Edward R. Hun I am permitted to give the following interesting case:

Case II. J. E. Lawyer, age 68, health always good except for the last few years, has had some difficulty in micturition; has never required medical aid until May 7th, when he suffered from retention of urine, which was relieved by catheterization. His urine dribbled away from the distented bladder without relief until May 11, when Dr. Hun was called. His abdomen was tympanitic with hiccough, rapid and feeble pulse; the left leg was œdematous and mottled up to the middle of the thigh; no induration could be felt in the course of the femeral artery. Through the aid of the catheter one quart of bloody urine was drawn off, followed by one ounce of clear blood which afforded great relief. May 12, no hiccough and less distress, the catheter was introduced but no urine followed; when withdrawn the eyes were obstructed by clots of blood; an injection was then used of warm water to clear the eyes of the catheter. Up to May 18th, the urine was drawn off three times per day and was much clearer, and the patient seemed bright and better. The œdema of the leg continued but is soft, and the superficial veins are more marked and are everywhere visible. He died May 21st.

Necropsy 24 hours after death. Head not examined. Thoracic and abdominal viscera normal except the bladder, which was distended with bloody urine and some firm clots of blood. All three lobes of the prostate were much enlarged and there was some ulceration at the upper and posterior portion of the middle or third lobe, from which the hemorrhage must have taken place.

Case III.* Has unusual interest from the circumstance that the subject was the eminent surgeon Dr. Alden March.

He had suffered many years from irritable bladder and hypertrophied prostate. Any unusual excitement such as the performance of a hazardous surgical operation was sure to increase this disturbance.

It is said that more than 15 years ago, while traveling with his

*Taken from the published report in the New York Medical Journal, by Dr. James H. Armsly. The Necropsy was written at Dr. Armsly's request by Dr. Edward R. Hun.

family in Switzerland, he met with an accident while descending Mount Righi; slipping on a rock and stricking the lower part of the abdomen on a projecting point.

The injury was severe, and confined him to his room in Lucerne several days with local symptoms. Before this time he provided himself with a urinal and when traveling kept it at hand night and day. Some time later he met with another accident in running up the steps of a railroad depot. Tripping on a step he struck heavily on the edge of the platform. He suffered very much at the time and was exceedingly alarmed. Speaking of the accident he said: "I thought that I had killed myself, that I had ruptured my bladder." He never recovered entirely from this injury.

A few weeks before his death he made the journey to New Orleans, to attend a meeting of the American Medical Association. On his way home he traveled night and day continually, which undoubtedly did him much harm, although upon his return he resumed his business and attended to distant calls.

After a fatiguing ride through rain and cold in the country, he returned home quite ill with fever and a constant desire to void urine. He used cathartics followed by opium together with warm baths, which afforded him relief for the time being, and from this attack he partially recovered and in a few days performed at the Hospital a difficult operation. From this time his health broke down and his decline was quite apparent, he was not enabled to do much more practice.

On the Sabbath, June 6th, he attended church and remained during the service, but was in great distress from the old trouble, and at the close of the service he rushed from his seat to the closet adjoining the lecture room and partially emptied his bladder.

He remarked to a friend, "I never suffered so much pain in all my life, I could not have borne it another minute."

From this time his professional labors ceased and he sought retirement at the house of his son-in-law's to avoid the annoyance of consultations.

The urine dribbled away an ounce or two at a time and no effort was made to introduce the catheter until some time after the 6th of June, some eleven days before his death, as he died upon the 17th of the same month, aged 74 years.

When an effort was made to introduce the catheter it was passed up its whole length without entering the bladder. Blood coagulated in the catheter, no urine passed through it, but some passed external to it and some followed its withdrawal. Again, a few days before his death another effort was made, when the patient was anesthetized the catheter was again passed its full length, when it met a firm resisting body, and again the effort was unsuccessful.

About this time symptoms of uræmic poisoning commenced, and finally hiccough, delirium and stupor set in, and the great surgeon closed his eyes in death, eleven days after he suffered so much while attending divine service

Autopsy—body well nourished; palpation of the abdomen revealed the presence of a hard globular tumor occupying the hypogastrium; the abdomen was laid open by a crucial incision; the abdominal walls contained a considerable layer of adepose tissue, and the muscles presented a healthy color; upon turning back the flaps, the bladder was found to be distended and occupied the hypogastric region from the pubes to the umbilicus, the fundus being a little to the left of the median line; there were some old adhesions between the omental and visceral peritonerium; a trocar was introduced into the anterior portion of the bladder, and rather more than a quart of slightly turbed urine was drawn off. A longitudinal incision having been made along its anterior walls, the internal surface of the bladder was brought into view and was found to present the reticulated appearance usually met with in cases where obstruction has been offered to the free flow of urine, but there was no abnormal thickening of the walls of the organ. A deep depression existed between the prostate, owing to the enormous hypertrophy of this gland. A catheter was now introduced through the urethra into the bladder without difficulty. The bladder with the prostate and a part of the membranous portion of the urethra was removed from the body in a mass, and the prostatic enlargement was now well shown, and appeared mainly due to hypertrophy of the two lateral lobes.

A catheter was passed from the bladder into the urethra until it merged externally, and then the incision already made along the anterior wall of the bladder was prolonged downward through the upper walls of the urethra, the catheter serving as a guide for the knife.

This having been done, it was found that, although the prostatic portion of the urethra was laid open, yet the membranous portion uncut. It was also observed that the connective tissue lying anterior to the prostate gland and neck of the bladder was stained and infiltrated with blood, although there was no evidence of an urinary infiltration. The middle lobe of the prostate was enlarged in such a manner as to form a cul-de-sac just below the vesical orifice of the urethra.

The kidneys were rather larger than usual, and contained several cysts filled with straw-colored fluid, which cysts were situated in the cortical substance and projected beyond the surface of the organ. The pelvis of both kidneys were enlarged, but whatever fluid they may have contained, escaped unnoticed when the ureters were divided. The renal tissue appeared somewhat congested, but was otherwise normal, and subsequent microscopic examination showed no alteration of the malpigian bodies or uriniferous tubules; the other abdominal viscera presented nothing abnormal. The head and thorax were not examined.

